# Organizational life cycle models: a design perspective

**DOI:** 10.1186/s41469-021-00090-7

**Published:** 2021-01-20

**Authors:** Luigi Mosca, Martina Gianecchini, Diego Campagnolo

**Affiliations:** 1 Centre for Systems Engineering and Innovation, Department of Civil and Environmental Engineering, Imperial College London, SW7 2BB, London, UK; 2 Department of Economics and Management “Marco Fanno”, University of Padova, Via del Santo 33, 35123 Padua, Italy; 3 Department of Economics and Management “Marco Fanno”, University of Padova and ICRIOS Bocconi University, Via del Santo 33, 35123 Padua, Italy

**Keywords:** Organizational life cycle, Organizational design, Vertical differentiation, Horizontal differentiation, Centralization

## Abstract

New competitive and environmental challenges have fostered renewed attention towards organizational design. This scenario calls for a significant return to organizational design studies that embrace a holistic approach, especially those focusing on the simultaneous interaction of multiple design elements. Organizational life cycle (OLC) models provide a fitting response to this call. In this paper, we review the organizational design characteristics of five seminal OLC models. We show that according to these OLC models, growth in size—which is described as unavoidable—generates business issues that firms are forced to solve by adopting only one possible organizational configuration, here following a deterministic organizational approach. We challenge this approach and propose conceiving of OLC as an evolutionary process, which calls for a variety of equifinal organizational solutions. We conclude by proposing future research avenues.

## Introduction

Early management scholars have recognized the importance of organizational design (e.g., March and Simon 1958; Burns and Stalker 1961; Lawrence and Lorsch 1967; Thompson 1967). Nevertheless, over the past several decades, the same literature has demonstrated a reduced interest in new research related to organization design. According to Greenwood and Miller (2010), this reduced interest is because of a shift in the level of analysis from the organization to the field, population and community; to the complex nature of today’s organizations demanding detailed, qualitative and time-consuming studies that do not align with actual publication pressures; and to an increasing interest in understanding the single dimensions of the organization (e.g., coordination mechanisms) rather than their interactions in the whole organizational configuration (Miller et al. 2009).

At the same time, new challenges have fostered renewed attention to organization design, such as globalization, outsourcing and capability development (Miller et al. 2009; Gulati et al. 2012; Van de Ven et al. 2013); indeed, Burton et al. (2020, p. 1) argue that ‘the field of organization design is undergoing a renaissance’. In this modern context, firms require fitting organizational designs (Galbraith 1999; Miller 2003) to renew their existing capabilities (Teece et al. 1997; Zollo and Winter 2002).

This scenario calls for a significant return to organization design studies that embrace a holistic approach (Meyer et al. 1993; Snow et al. 2005), focusing on the simultaneous interactions of multiple organizational design elements. Organizational life cycle (OLC) models provide a fitting response to this call.

OLC models consider a firm’s life to be a sequence of different developmental stages. Developed between the 1960s and 1990s, the most relevant OLC models shared the organism life cycle analogy proposed by Gardner (1965). Indeed, like people and plants, organizations ‘have a green and supple youth, a time of flourishing strength, and a gnarled old age’ (Gardner 1965, p. 20). A central tenet of life cycle theory is that organizations move through a series of phases. Hanks et al. (1993, p. 7) defined a life cycle phase as ‘a unique configuration of variables related to organization context or structure’. Therefore, the OLC includes a sequence of events that describe how things change over time (Van De Ven 1992), a hierarchical progression that is not easily reversed and a composite of a broad range of organizational activities and structures (Quinn and Cameron 1983). In short, OLC models simplify a myriad of facts associated with transformational change, reducing the complexity to a uniform, appealing, predictable and deterministic pattern (Stubbart and Smalley 1999).

Researchers have tested the empirical validity of these models (e.g., Dodge and Robbins 1992; Lester et al. 2003; Primc et al. 2020), and they have also applied these models as guiding frameworks for studying the development of specific managerial practices (e.g., human resources management, corporate governance), reaching only partially conclusive findings (see, e.g., Jawahar and McLaughlin 2001; Kallunki and Silvola 2008; Wang and Singh 2014). Despite these contributions, an organizational design inquiry into such models is still missing.

Therefore, building on the five primary elements of good theory (i.e., why, when, who, what and how) suggested by Whetten (1989), we (1) review five seminal OLC models—Lippitt and Schmidt (1967), Greiner (1972), Adizes (1979), Galbraith (1982) and Churchill and Lewis (1983)—proposing an in-depth analysis of their organizational design characteristics, and we (2) discuss the relevance of the OLC perspective for describing the evolution of the firms in the actual business environment. By reviewing the seminal OLC models through Whetten’s five primary elements of good theory, we not only extend Levie and Lichtenstein’s (2010) analysis, which was limited to three theoretical elements (what, how and why), but we also add to previous reviews on OLC models (e.g., Phelps et al. 2007; Muhos et al. 2010; Muhos 2015; Tam and Gray 2016; Jirásek and Bílek 2018), which have neglected to analyze the organizational design characteristics inherently associated with each stage of the models (namely vertical and horizontal differentiation, coordination mechanisms, centralization and decentralization, standardization and mutual adjustment).

Overall, our analysis demonstrates that the OLC models propose a deterministic trajectory of organizational development showing limited explanatory power when confronted with the challenges of the actual business environment. Therefore, we propose of conceiving of the OLC as a process in which the ‘engine’ is the changes associated with the variety and uncertainty of the environment: addressing those changes, companies evolve independently from their size following unpredictable paths with a variety of equifinal organizational configurations.

The remainder of the current paper is organized as follows: the next two sections describe the research methodology and illustrate the selected OLC models. Then, we present an analysis of the organizational design characteristics of the models through the five primary elements of good theory. Finally, we discuss the limitations and ongoing relevance of the OLC perspective in the actual business environment.

## Research methodology

Our literature review focuses on the OLC models published in management journals and considers three steps. First, to provide a revised and up-to-date overview of the OLC models, we searched the ‘ISI Web of Knowledge’ database (time span: 2000–2020 and Social Sciences Citation Index) using the following keywords: ‘review life cycle of organization’ and ‘review organizational stages and growth’. This search produced six review articles: Phelps et al. (2007), Levie and Lichtenstein (2010), Muhos et al. (2010), Muhos (2015), Tam and Gray (2016) and Jirásek and Bílek (2018).

Second, using the snowball approach, we analyzed the OLC models presented in the six reviews. Then, we selected the OLC models meeting the following three criteria: (1) the model should be novel and not based on previous models; (2) the model should present and discuss how organizational design characteristics change in firms’ life cycles and (3) the model should be an original intellectual source and not only an empirical test. As a result, we excluded the papers that adopted OLC models to study managerial problems not related to organizational design, including, for instance, Koberg and colleagues (1996) and Kallunki and Silvola (2008), both of which use Greiner’s model to study, respectively, the organizational innovation and the use of activity-based costing in firms’ life cycles. Through this analysis, we selected five models: Lippitt and Schmidt (1967), Greiner (1972), Adizes (1979), Galbraith (1982) and Churchill and Lewis (1983).

Third, both of the literature reviews used during the first step of analysis considered articles and contributions published between 1960 and 2006. Therefore, we ran a search in the ‘ISI Web of Knowledge’ database, selecting the time span of 2006–2020. To search for other OLC models, we chose the same keywords adopted by previous reviews: life cycle growth, stages theory of growth, stages of organizational growth and organizational life cycle model. We then applied the three criteria for selecting new models but without success; we did not discover any other model. Therefore, we continued our analysis based on the five previously mentioned models.

## Organizational life cycle: a description of the most relevant theoretical models

To provide an overview of the five models, we briefly describe each one. Then, through the information-gathering questions, we focus on their organizational design characteristics.

### Lippitt and Schmidt (1967) model

Focusing on the private sector, Lippitt and Schmidt (1967) developed one of the earliest OLC models. They suggest that firms progress through three stages of development, facing six major ‘managerial concerns’ to progress from one stage to the next. At *birth,* critical concerns include the creation of the system and achieving a survival threshold. During *youth,* the main concerns are stability and reputation. During *maturity*, achieving uniqueness and responding to diverse societal needs become major concerns. Management must solve the crises in a way that creates a sound base for dealing with future crises. When an issue is solved, firms progress to the following stage. Failures occur when managers fail to recognize the significant crises arising in the organizational life cycle. According to the authors, most companies retain, often by preference, simple organizational structures, uncomplicated product programs and ordinary ambitions.

### Greiner (1972) model

Greiner (1972) assumes that a firm’s life unfolds through a sequence of five stages of *evolution* and *revolution*. A stage of *evolution* is a period of growth where no major upheaval occurs in organizational practices. In contrast, a *revolution* is a period of substantial turmoil in an organization’s life. The resolution of each revolutionary period provides the go-ahead to move onto the next stage. Greiner (1972) describes the growth stages based on five parameters: management focus, organizational structure, top management style, control system and management reward emphasis. The growth stages include the following: (1) creativity-led growth is broken off by a crisis of leadership; (2) direction-led growth is broken off by a crisis of autonomy; (3) delegation-led growth is broken off by a crisis of control; (4) coordination-led growth is broken off by a crisis of bureaucracy or a red-tape crisis; and (5) collaboration-led growth is broken off by a crisis of lack of internal solutions for growth. Evolutionary periods range from 4 to 8 years depending on the industry: in fast-growing industries, the periods may be shorter, while in mature industries, the periods may be longer.

### Adizes (1979) model

Adizes’ (1979) model suggests that firms move through stages because of changes in emphases on four activities: producing results (P), acting entrepreneurially (E), administering formal rules and procedures (A) and integrating individuals into the organization (I). As the organization passes from one phase to the next, it emphasizes different roles, and the resulting role combinations produce varying organizational behavior. Organizational decline occurs primarily because of an overemphasis on bureaucracy, rules and procedures. The model suggests that organizations develop through 10 stages: courtship, infant, go-go, adolescent, prime, mature, aristocratic, early bureaucracy, bureaucracy and death. Progression across stages occurs mainly by overcoming the growth problems of successive stages. Organizations begin with an emphasis on entrepreneurial activity that later becomes coupled with an emphasis on producing results.

### Galbraith (1982) model

The model developed by Galbraith (1982) intends to capture the predictable dynamics of a new organization’s stage-wise development; the basic idea is that firms move through predictable stages, but according to the author, managers do not think in a stage-wise manner, despite the predictability of these stages. His model focuses on start-up ventures. These companies develop a business idea that consists of a market to be served, products to be sold, the basis for dominating the niche and the resources and resource combinations needed to achieve dominance. Galbraith’s (1982) model involves five stages: proof-of-principle prototype, model shop, start-up volume production, natural growth and strategic maneuvering. To pass from one stage to another, the firms have to increase in size. Moreover, growth is guided by the product market and related to the product life cycle.

### Churchill and Lewis (1983) model

Churchill and Lewis (1983) used a combination of empirical research and a review of previous theoretical works to develop a new OLC model. Their theoretical development derives from the identification of three weaknesses in previous models. First, previous models assume that a company must grow and pass through all the stages of development or die during their attempt to do so. Second, a company is unable to capture the important early stages in a company’s origin and growth. Third, they define company size mainly in terms of annual sales (although some mention the number of employees) while ignoring other factors, such as value added, number of locations, complexity of product line and rate of change in products or production technology. As a consequence, the model proposes five stages: conception/existence, survival, profitability and stabilization/growth, take-off and maturity. Each stage is characterized by an index of size, diversity and complexity, as described by five management factors: managerial style, organizational structure, extent of formal systems, major strategic goals and the owner’s involvement in the business. The model focuses on small enterprises. To grow and increase in size and profitability, firms must adapt to the environment.

## An analysis of the main features of the OLC models

To analyze and compare the main features of the selected OLC models, we discuss all five elements (i.e., why, when, who, what and how) proposed by Whetten (1989) as the primary elements of good theory. The ‘what’ question provides the factors that must be considered in explaining the phenomena under study. The ‘how’ of a theory demonstrates the relationships between the identified factors. The ‘why’ element explains the selection of factors and the proposed causal relationships. The ‘who and when’ questions validate theory with empirical data while setting limits on its uses and applications.

Adapting these insights to our analysis, we develop the following five questions:

- Why: why do firms move from one stage of development to the next (i.e., analysis of the internal and external pressures to change)?
- When: what is the duration of each stage, and what are the variables used in defining the organizational evolution within each stage?
- Who: who are the actors managing the organizational development?
- What: what are the organizational design features that characterize the firm during each stage?
- How: how do firms move from one stage to the next?

Because the present article focuses on the organizational design aspects characterizing the different stages of development, we thoroughly discuss the relevant ‘what’ questions in a specific section of the article. In the following paragraphs, we analyze the other four elements (Table 1).

**Table 1:** Description of the five OLC models

Author(s)	Numbers of stages	Why	When	Who	How
Lippitt and Schmidt (1967)	Three: birth, youth and maturity	Both external and internal	Flow of time (one of the issues will acquire exceptional importance)	Management	Solve the crisis and create the base for the future crises
Greiner (1972)	Five: creativity, direction, delegation, coordination and collaboration	External	Both age and time have to increase	Top management	Solving the revolution
Adizes (1979)	Ten: courtship, infant, go-go, adolescent, prime, mature, aristocratic, early bureaucracy, bureaucracy and death	External	In the long run, organization must adapt to its external environment	Management	Change role combination and organizational behavior
Galbraith (1982)	Five: proof-of-principle prototype, model shop, start-up volume production, natural growth and strategic maneuvering	External (market-product life cycle)	Increase in size	Management	Implement the right organization that fits with the size
Churchill and Lewis (1983)	Five: conception, survival, profitability, take-off and maturity	Both external and internal	Increase in size	Business owner, management	Firms have to increase in size and profitability

### Why: the pressures to change

Internal and/or external factors explain why companies change their organizational structure and move from one stage to the following one. Internal factors include strategic and managerial decisions, while external factors include market and competitive pressures.

Lippitt and Schmidt (1967) and Churchill and Lewis (1983) consider both the external and internal pressures in motivating organizational evolution. These factors affect different phases of the organizational life cycle: initially, firms confront external pressures to affirm themselves and survive in the competitive market. Then, they face internal issues related to the organizational structure and management of human resources. According to Greiner (1972), the transitions across stages are mainly determined by internal factors: the ‘revolution’ moments are indeed determined by changes in firm strategy, managerial objectives and/or issues in organizational structure.

A different perspective has been adopted by Adizes (1979) and Galbraith (1982), both of whom consider only external pressures. According to Adizes, firms have to adapt to their external environment to grow: for instance, during the first stages of the OLC, organizations can survive in the market by increasing their sales and, therefore, responding to customers’ needs. Similarly, Galbraith focuses on market share as a means of sustaining firm growth and profitability.

### When: the length of the stages

The second question concerns the ‘unit of measure’ adopted by the OLC models to describe the length of each stage. The models do not explicitly indicate a time length for the stages, and in some cases, they associate the duration of the stage with the size of the firm.

Though they do not indicate a number of years for each stage, Lippitt and Schmidt (1967), Greiner (1972) and Adizes (1979) refer to the relevance of the time in their models. According to Lippitt and Schmidt, time is relevant because organizational issues may become significant crises if they are not resolved within a reasonable time frame. According to Greiner’s model, as time flows, new and different organizational problems emerge: the combination of age and size exacerbates the problem, activating a revolution period. Adizes suggests that during each life cycle stage, a typical pattern of entrepreneurial and management behavior emerges; therefore, time is relevant in predicting companies’ activities.

Whereas previous models consider the flow of time as the most relevant factor in explaining the OLC model structure, Galbraith (1982) and Churchill and Lewis (1983) focus on the organization’s size. As a consequence, size, not age, indicates the company’s life cycle stage. In particular, Galbraith claims that firm growth is driven by the growth of the market, and then, each phase depends on external resources. When managers find the right way to govern and exploit external resources, the firm moves to the next stage. Churchill and Lewis (1983) relate firm growth to profitability: when the latter is satisfactory, the firm moves from one stage to the next.

### Who: the actors leading the organizational development

Concerning the actors who lead the organization’s development along its life cycle, all five models generically indicate that management is primarily responsible, namely the executives and/or founders. In particular, managerial responsibilities include recognizing the organizational issues when they emerge, solving problems and determining the appropriate configuration of organizational design elements to move from one stage to the next.

The five models fail to explain how a management team either supports or substitutes for the firm’s founder, but they predict when this process occurs. For example, Lippitt and Schmidt (1967) assert that firms have entrepreneurs and a management team in the first stage. Together, they make key decisions for their organizations, such as how much risk to take. Greiner (1972) predicts that a business manager will be hired in the second stage; thus, in the first stage, only the founder(s) manages the firms. Adizes (1979), Galbraith (1982) and Churchill and Lewis (1983) claim that a management team appears in the third stage to support the founder in managing new departments and information and control systems. In essence, the five models do not focus on how a management team flanks the firm’s founder; however, they predict that the latter is not able to manage the growth of the firm alone.

### How: the process of development

The process that sustains the development of the organization along its life cycle varies significantly in the five models. The OLC models by Lippitt and Schmidt (1967) and Adizes (1979) identify the predictability of ‘crises’ as the key elements activating the process of organizational development. According to Lippitt and Schmidt, managers have to constantly monitor the market to identify ‘potential problems’ (such as market uncertainties and creditor demands). Adizes asserts that long-range planning is necessary to anticipate and manage future endeavors, markets and technologies. Therefore, firms can move to the next stage only if managers make decisions at the right time and with the right intensity.

The ‘revolution periods’ described by Greiner (1972) are phases of considerable organizational turmoil (e.g., demand from middle managers for greater autonomy and the need for new, motivated employees). In this model, the nature of the solutions implemented by managers determines whether firms will move forward to the next stage.

The OLC models proposed by Galbraith (1982) and Churchill and Lewis (1983) consider organizational growth (in size) as the driving mechanism for development. The former claims that managers should define the right combination of all resources (such as people, rewards and structure) to manage growth in each stage. The latter affirms that firms acquire resources to move to the following stage when they increase their market penetration, economic success and profitability.

## The ‘what’ of the OLC models: organizational design characteristics

The *what* question concerns firms’ organizational design characteristics in different stages of their life cycles. All the models agree in suggesting that in the early stage of development, companies lack organizational structure. When the firm is created, owners manage the business, and they are simultaneously entrepreneurs and managers (Adizes 1979). The business owner (Greiner 1972; Churchill and Lewis 1983) deals with issues in business ideas and product development (Lippitt and Schmidt 1967; Galbraith 1982). Delegation is low, and the company is not structured. As a result, organizational issues will emerge as the company, after surviving the start-up phase, tries to move to a further stage of development.

Table 2 summarizes the results of our analysis concerning some relevant organizational design parameters in each phase of a firm life cycle: vertical differentiation, horizontal differentiation, coordination mechanisms, centralization and decentralization and standardization and mutual adjustment.

**Table 2:** Organizational characteristics of the OLC models

	Lippitt and Schmidt (1967)	Greiner (1972)	Adizes (1979)	Galbraith (1982)	Churchill and Lewis (1983)
Vertical differentiation		The number of supervisors increases up to fourth stage and decreases in the fifth stage (from 2° to 5°)		Venture managers manage the span of control issues (from 2° to 5°)	Balance between number of employees and supervisors (from 2° to 5°)
Horizontal differentiation	Functional and divisional structure (2°, 3°)	Functional to divisional, line-staff and then to matrix structure (from 2° to 5°)	Functional then divisional structure (from 2° to 9°)	Functional to modified functional and then to divisional or matrix structure (from 2° to 5°)	Functional, a divisional and line/staff structure (from 3° to 5°)
Coordination mechanisms	Systematic plans and upward communication flows (2°, 3°)	Budgets, profit centers and task group (from 2° to 5°)	Organizational culture and facilitators (from 2° to 9°)	Structures to coordinate, planning and budgeting systems (from 2° to 5°)	Budgets, operational and strategic planning (from 3° to 5°)
Centralization/decentralization	C: venture managers do everything (1°) D: delegation increases over time (2°, 3°)	C: centralization to have control and coordination (1°, 4°, 5°) D: functional organization with decentralization (2°, 3°)	C: founders have all the power (from 1° to 4°) D: decentralize to the subordinates (from 5° to 9°)	C: owner has all the power (1°, 2°, 3°) D: the management decentralize to the departments (4°, 5°)	C: the owner does everything (1°, 2°) D: decentralize to functional manager (3°, 4°,5°)
Mutual adjustment/standardization	M.A.: firms should move with speed and flexibility (1°) S.: firms implement and then update policies (2°, 3°)	M.A.: informal communication, team action, social control and self-discipline (1°, 5°) S.: formal communication and planning procedures (2°, 3°, 4°)	M.A.: people take own initiative (1°, 2°, 3°) S.: formal plans and written procedures (4°, 5°, 6°, 7°)	M.A.: decision process is spontaneous (1°, 2°) S.: formal rules and control (3°, 4°, 5°)	M.A.: no formal planning (1°, 2°) S.: formal systems (3°, 4°, 5°)
Organizational structure	Functional and divisional	Functional, divisional, line-staff and matrix	Functional and divisional	Functional (and with integrated departments), divisional and matrix	Functional, divisional, and line-staff

### Vertical differentiation

Vertical differentiation involves the installation of a chain of command among employees and managers. Thus, it relates to the number of supervision levels (Hall et al. 1967; Meyer 1968). Vertical differentiation is analyzed at different levels of detail, meaning that some models explicitly address this issue while others ‘implicitly’ refer to an increased number of hierarchical levels as companies evolve. Concerning the latter perspective, Lippitt and Schmidt (1967) predict that during the shift from the first to second stage, the organization becomes taller. By contrast, the last stage requires a flat organizational structure. However, the authors do not provide a detailed description of how these changes occur. Similarly, Adizes (1979) discusses relevant issues regarding the development of hierarchy (i.e., decentralization of power), but he does not define how the organizational structure develops over time.

On the contrary, the other three models clearly describe changes in the vertical structure. In particular, Galbraith (1982) argues that vertical differentiation is initially related to issues of coordination and the supervision of new employees hired in the second and third stages: the owner should add levels between him- or herself and new entrants to manage the increased span of control. Then, in the last two stages, the owner hands over decision-making power to product managers who can deal with the matter of diversity (new products and functions). Similarly, Churchill and Lewis (1983) claim that the development of the hierarchical structure relates to the necessity for more supervisors as the firm size increases: when an organization becomes larger, an effective delegation process and greater number of managers allow the company to preserve its ability to make innovative decisions. In contrast, Greiner (1972) declares that the number of supervisors increases up to the fourth stage but decreases in the fifth stage.

Ultimately, the three models that describe the development of vertical differentiation assert that the organizational hierarchy becomes taller over firms’ life cycles. Only Greiner (1972) predicts an initial rise in organizational hierarchy followed by a decrease in the last stage.

### Horizontal differentiation

Horizontal differentiation is explored in detail by all five analyzed models. In general terms, the models agree on depicting a trajectory of organizational development that is initially based on a functional criterion of horizontal differentiation followed by a divisional one. In particular, activities are grouped together by common functions from the bottom to the top of the organization in terms of functional structure. Each functional activity, such as accounting, engineering, human resources and manufacturing, is grouped into a specific department (Taylor 1947). The divisional structure instead occurs when departments are grouped together based on organizational outputs. The divisional structure is sometimes organized by product line(s) or profit centers (Anand and Daft 2007).

According to this trajectory of development, Lippitt and Schmidt (1967) explain that firms first adopt a functional structure, with a key function represented by the research and development department. Then, when firms enter the maturity phase, a divisional structure—specialization in products or services—is adopted. Similarly, Adizes (1979) claims that developing firms need a directive board to plan the organization structure in advance. First, a functional structure is adopted; then, to serve new products and markets, the organization moves towards a divisional structure in its markets, products or profit centers. Such an organizational form stimulates and develops the entrepreneurial personality of the managers. If the divisional structure is not well adopted, the company fails.

The other three OLC models support steps of organizational development other than what is found in the divisional structure. Combining the two horizontal differentiation criteria, these three OLC models suggest that companies first adopt a line-and-staff and then a matrix structure. The line-and-staff organization combines the line units, namely all the activities directly related to organizational goals (either functions or divisions), with staff departments that support and advise the line departments (Fayol 1949). The matrix combines a vertical structure with an equally strong horizontal overlay. Although the vertical structure provides traditional control within functional departments, the horizontal overlay provides coordination across departments to achieve profit goals. This structure has lines of formal authority along two dimensions, such as functional and product or product and region (Mee 1964; Galbraith 1971).

Greiner (1972) asserts that as firms grow, a functional structure is introduced to separate manufacturing from marketing activities. Then, as firms become larger, the increased delegation goes hand in hand with adopting either a divisional or line-and-staff structure. The divisional structure focuses on market territories, while the line-and-staff structure combines product units with staff departments. Finally, in the last stage, Greiner (1972) suggests implementing a matrix structure to assemble teams for addressing specific problems and solve possible conflicts between the line-and-staff.

Similarly, Galbraith (1982) affirms that developing firms should use a functional structure to coordinate new specialized product workers when they are hired. Then, more organizational units (functions) are added to manage the increased production volume. If firms assume a product differentiation strategy, they satisfy the need to combine functional teams and product managers by ‘integrating departments’. In the last stage, firms can adopt either a divisional structure (creating profit centers around regions, products or markets) or a matrix structure to solve the issues related to diversification and vertical integration.

Finally, Churchill and Lewis (1983) suggest that firms require a functional structure to manage their financial, marketing and production activities. Then, firms should be organized in either sales or production groups (divisional structure) to face issues related to the maintenance of managerial effectiveness in a rapidly growing organization. When firms become larger, they require a line-and-staff structure to remain flexible and improve the managers’ entrepreneurial spirit.

To sum up, the authors claim that when firms grow and employee numbers increase, the owners cannot manage everything alone; they need to set up a differentiated organizational structure. The first suggested arrangement is a functional structure. Then, they propose a similar organizational development through divisional, line-and-staff and matrix structures.

### Coordination mechanisms

March and Simon (1958) claim that coordination mechanics relate to a division of work that causes interdependence among organizational units. According to the OLC models, the need for coordination mechanisms emerges together with changes in the horizontal differentiation criteria. To manage such issues, the authors suggest different mechanisms.

Lippitt and Schmidt (1967) propose managing increasing complexity because of the addition of new departments with systematic plans and long-range planning. Furthermore, the authors promote the adoption of upward communication systems that can share information between departments.

Greiner (1972) and Churchill and Lewis propose different mechanisms to address specific issues: budgets should support coordination when functions are created; profit responsibility is introduced to coordinate and stimulate employees who belong to different divisions; teams and task groups must satisfy the need for cross-functional integration; and strategic planning and standard cost systems should reduce inefficiencies generated by the increasing size.

Galbraith (1982) asserts that having a hierarchy can improve coordination and control when new departments are added. He claims that general management (e.g., multifunctional managers) can solve conflicts among functional units. When firms increase their number of products, cross-functional teams are required. Finally, if firms pursue growth through diversification by regions, products or markets, managers should combine the use of profit centers and corporate culture to coordinate employees.

Adizes (1979) divides the life cycle of firms into two main periods: before and after maturity (the sixth stage). According to the author, up to maturity, employees are guided by an *internal agent *(expert individuals working for the organization) and are oriented by the organizational culture. After the maturity stage, firms need an *external agent of change* (outside consultants who are temporarily employed by the organization) to lead and coordinate workers.

In summary, the authors affirm that firms should set up both the organizational structure and coordination mechanisms at the same time. The analysis shows that there is a lack of agreement regarding which coordination mechanism best fits each type of organizational structure.

### Centralization and decentralization

Centralization and decentralization define the distribution of power and level of participation in strategic decisions within an organization (Hage 1980). For various reasons, issues of centralization and decentralization emerge during the life cycle of firms.

Whereas Adizes (1979), Churchill and Lewis (1983) and Lippitt and Schmidt (1967) explain that decentralization is adopted to motivate employees to follow their own initiatives and attract creative workers when the size of a firm increases, Galbraith (1982) suggests increasing decentralization to support product diversification, hence assigning managers the responsibility of new products.

Different from the other models, Greiner’s model (1972) claims that growing firms should decentralize to satisfy the demand for greater autonomy from middle managers; however, when firms reach their largest size, namely in the last two stages, centralization becomes necessary again to regain control and achieve greater coordination over firms.

In brief, the authors affirm that the process of decentralization is directly linked to the growth of the firms: bigger firms need more delegation. According to the authors, decentralization allows firms to achieve diverse benefits, such as increased worker motivation and greater work flexibility. Only Greiner (1972) holds an opposing view: he claims that centralized management is the best choice to resolve issues that appear with large firms.

### Standardization and mutual adjustment

All the authors consider the degree of formalization of the operational processes and separate between standardization and mutual adjustment, which are at the opposite extremes of a continuum. Standardization is a way of using rules and norms to standardize workers’ behavior, while mutual adjustment is the process through which employees use their judgement rather than standardized rules to address problems, guide decision-making and promote coordination. Lippitt and Schmidt (1967) suggest implementing and then updating administrative policies in the second stage. Adizes (1979) claims that in the maturity (sixth) stage, a well-managed bureaucracy is essential for firm survival. Galbraith (1982) and Churchill and Lewis (1983) recommend adopting formal rules in the third stage to have a better control system and improve efficiency in strategic planning.

In contrast, Greiner (1972) maintains that when firms reach the fifth stage, they emphasize greater spontaneity in their management action. Therefore, as in the first stage, employees’ social control and self-discipline take over for the formal control that was used up to the previous stage.

Overall, the five models predict that small firms do not need to standardize job activities in the early stages of their life cycle. When the number of workers, departments and functions increases, firms should standardize procedures and routines. Greiner (1972) does not completely agree with this point; indeed, he maintains that the last stage of firms is based on manager flexibility and spontaneity.

## OLC models: empirical validation

In this section, we explore whether and how the selected five OLC models have been empirically validated. To do so, we searched the ‘ISI Web of Knowledge’ database and manually reviewed all the empirical studies that cited such models.

Overall, it emerges that organizational scholars have mostly utilized the OLC models to leverage their theoretical assumptions (e.g., firms grow over time) in a variety of domains (family businesses, circular economy) and industries (e.g., service, engineering, building, healthcare, media, chemicals, finance) or to explore specific managerial practices (e.g., human resources management, corporate governance) (Dodge and Robbins 1992; Jawahar and McLaughlin 2001; Lester et al. 2003; Kallunki and Silvola 2008; Brettel et al. 2010; Wang and Singh 2014; Primc et al. 2020).

More limited are the studies have tested their predictions about a firm’s stages of development (Phelps et al. 2007; Levie and Lichtenstein 2010; Muhos 2015). In particular, we found three studies (i.e., Tushman et al. 1986; Eggers et al. 1994; Sukova 2020) aimed to conduct an empirical validation of only two of the OLC models analyzed in the present study, namely Churchill and Lewis (1983) and Greiner (1972).

The study by Eggers et al. (1994) tests Churchill and Lewis’ (1983) model through the exploration of the development of a sample of small firms that were randomly selected for geographical location and industry. The authors find substantial support for the model. However, even if most of the firms follow predictable stages of development and adopt similar organizational forms in different stages of their life cycle, considerable variability still remains.

More recently, Sukova (2020) conducts an empirical validation of Greiner’s (1972) model by studying six large automotive firms based in the Czech Republic. The author finds empirical validation for Greiner’s (1972) model by showing that large firms increase vertical differentiation and centralization in the third and fourth stages of firms’ lives. Previously, Greiner’s (1972) model inspired the study by Tushman et al. (1986) on a large sample of firms of different sizes (both small and large firms), operating in different industries and located in different countries. The authors, however, do not find empirical evidence for this model, showing that there are no general patterns in the sequence of frame-breaking changes and growth stages.

The limited empirical support for the predictions of the OLC models casts doubts about their suitability in describing companies’ development. As a consequence, in the following section, we discuss their relevance to the actual business environment.

## OLC models: present and future

The results of the literature review demonstrate that OLC models advance how growth in size—which is considered unavoidable—is linear and sequential and generates business issues that the firm is forced to solve by adopting a predetermined sequence of organizational configuration (Quinn and Cameron 1983; Stubbart and Smalley 1999; Rutherford et al. 2003). In particular, as companies become larger and older, they move from a simple/entrepreneurial structure to a functional and then a divisional structure. The reference to such organizational models is (most likely) intentionally loose and vague: for instance, Churchill and Lewis (1983) refer to a ‘line and staff’ organization as the last stage of organizational development. Providing a limited amount of details about the actual structures adopted by companies in the different stages, the authors of the OLC models open further avenues of research about the organizational complexity hidden behind those simplified and generic labels. In addition, it is worth noting that all the models considered in our analysis were published by American scholars to describe their national business context, which between the late 1960s to the early 1980s was characterized by a positive cycle and steady economic growth (Hodrick and Prescott 1997). Today’s business environment is instead characterized by volatility, uncertainty, complexity and ambiguity (Whiteman 1998; Bennett and Lemoine 2014). The international economy is highly interconnected, and sudden and unpredictable shocks may upset the markets where companies are competing, challenging their possibility of growing or even surviving. In such situations, firms must quickly frame the cause of the crisis, identify the most appropriate organizational structure and adjust accordingly while preserving the evolutionary capabilities for the next stage.

In this *new* setting, growth in size cannot be taken for granted. First, companies can remain stable in their size while growing in their other relevant business dimensions, such as relationships and capabilities (Furlan and Grandinetti 2011; Nason and Wiklund 2018). Second, during their life cycles, companies are more likely to sustain phases of growth that could be followed by phases of rightsizing (associated with shocks and crisis management) instead of continuous growth. Therefore, we sustain that the OLC of a company is better conceived as an evolutionary process instead of a sequence of growth (in size) stages: companies characterized by structural inertia (Hannan and Freeman 1984) and, therefore, inherently resistant to changes proceed in their life cycle continuously adapting—sometimes successfully and sometimes not—to relevant internal and external changes. Because these changes are scarcely predictable, they are likely to affect companies to a different degree, reducing the explanatory power of universal prescriptive OLC models.

Notwithstanding these limitations, we believe that the OLC perspective might preserve its validity if further developed along the trajectory of an enhanced understanding of the interdependences across the why, when, who, how and what. As a consequence, we discuss the OLC perspective that addresses Whetten’s five questions in the actual business environment. In particular, in the remaining part of this section, we explore what could be the organizational design consequences (the ‘what’) of the *current* why, when, who and how of a firm’s evolutionary process. Establishing the appropriate levels of vertical and horizontal differentiation, identifying effective coordination mechanisms and deciding the level of centralization and formalization are challenges that are constantly renewed over the evolution of the firm and that hardly find a unique (deterministic) answer.

As for the answer to ‘why’ firms evolve, traditional OLC models suggest that both external (e.g., market share, customer needs) and internal (e.g., strategic objectives, human resources management issues) factors trigger organizational development. These forces are still relevant in the actual business environment; however, when the OLC models were developed, business volatility and uncertainty were lower, while nowadays, markets appear less predictable. Therefore, strategic and operating planning activities aimed at sustaining firms’ growth may suffer from a lack of effectiveness if they cannot adapt to changing conditions. To be ready to react to such reduced predictability, firms need to find a right balance between standardization and mutual adjustment, most likely favoring the latter. For instance, one of the consequences related to an unforeseen and unpredictable event such as COVID-19 is the diffusion of remote working: estimates from Eurofound (2020) suggest that close to 40% of those currently working in the European Union began to telework full time as a result of the pandemic. Some big companies all over the world (e.g., Facebook) are evaluating the adoption of remote working as the standard way to organize employees. Many firms were ill-prepared to manage this change because remote working used to be marginal: in 2019, 5.4% of employed people in the European Union usually worked from home, and that percentage has been constant throughout the last decade. Hence, standardized procedures may be of limited effectiveness to sustain an organizational development that must face the growing and emerging demands related to remote working (Tietze and Musson 2005). On the contrary, organizations may lean on mutual adjustments to redesign workplaces and redefine time schedules according to the workers’ needs.

As the question about ‘when’ is concerned, our literature review demonstrated that OLC models suggest that transitions from one stage to the next are related to a combination of ageing and growing: as companies become older and larger, new management issues emerge, and they are forced to proceed along their natural evolutionary pattern. Even if the duration of the stages and their relationship with the organization’s chronological age are debated (Bailey and Grochau 1993; Rutherford et al. 2003), all the models implicitly adopt a linear perspective about growth in size. In today’s business environment, the lifespan of firms is shorter, and firms’ growth may be exponential. A recent study by McKinsey (2019) found that the average lifespan of companies listed in the Standard and Poor’s 500 was 90 years in 1935, 61 years in 1958, 35 years in 1970, and today less than 18 years. Young firms are more profitable compared with the older ones: companies in the Standard and Poor’s Global 1200 that were founded within the past 30 years generated four times as much shareholder value as longer-standing companies. However, such growth is not a guarantee of survival in their actual form because the same McKinsey (2019) report estimates that in 2027, 75% of the companies currently quoted on the Standard and Poor’s 500 will have disappeared because they will be bought-out, be merged or go bankrupt. In this competitive context, companies are required to be *agile* to successfully manage uncertainties (Sull 2009) and to be *resilient* to react to crises and adversities (Linnenluecke 2017; Gubitta and Campagnolo 2020). Organizational agility can be defined as ‘the capacity of an organization to efficiently and effectively redeploy/redirect its resources to value creating and value protecting (and capturing) higher-yield activities as internal and external circumstances warrant’ (Teece et al. 2016, p. 17). Similarly, organizational resilience refers to the ‘maintenance of positive adjustment under challenging conditions’ (Sutcliffe and Vogus 2003, p. 95) and is usually articulated as bouncing back from adversity (Williams et al. 2017) and as having the ability to ‘anticipate, avoid, and adjust to shocks in their environment’ (Ortiz-De-Mandojana and Bansal 2016, p. 1615). Companies can foster their agility and resilience, balancing the number of hierarchical levels and degree of centralization of decision-making processes. Indeed, flat and decentralized structures ‘where employees are given significant autonomy in how to carry out their work or which projects to undertake’ (Billinger and Workiewicz 2019, p. 17), such as collaborative organizational forms (Kolbjørnsrud 2018) are more agile and resilient than vertical and centralized structures. A trend toward a flat organization has been reported by several scholars (Cunha et al. 2011; Foss and Dobrajska 2015; Lee and Edmondson 2017; Burton et al. 2020). This evidence notwithstanding, other scholars (Sanner and Bunderson 2018) contend that in the case of team-based organizations, hierarchy is essential for helping a team engage in and get the most out of its efforts to learn and innovate. Burton et al. (2017) claim that even novel organizations—see, for instance, the case of GitHub—redesign their structure to introduce traditional hierarchical characteristics, thus showing that in novel (growing) organizations, the hierarchy preserves its role. These opposite views are the consequence of the contested nature of the hierarchy in today’s competitive setting, calling for a better understanding of how vertical differentiation and decision-making processes are executed. A recent contribution of Romme (2019), while providing a systemic perspective on the argument, defines organizational hierarchy ‘as a sequence, or ladder, of accountability levels’ (p. 8). As a ladder of accountability levels, hierarchy can be seen both as a chain of command, that is, as a ladder of decision-making authority levels (in line with the mainstream view of top-down organizational structures) and as individuals taking responsibility for higher-level tasks, that is, as a ladder of self-organized responsibility levels [in line with the recent trend of flat, bottom-up organizational structures, such as a holacracy (Robertson 2016)]. The difference between the two approaches resides in the distinction between *authority* and *responsibility*. Although *authority* deals with the ‘power to decide’ and is externally assigned, *responsibility* deals with ‘getting the job done’ and is somehow internally driven because it derives from an intrinsic obligation that individuals feel. Responsibility ladders are emergent in nature, thus showing a better fit with dynamic environments that require continuous evolution. Conversely, authority ladders are planned, thus adjusting better to stable environments that call for control and predictability. As a consequence, the presence of both ladders of accountability would be synergistic because they simultaneously promote adaptation and control. According to the analysis of mainstream OLC models, we can assert that ladders of responsibility are typical of new ventures or small and medium enterprises (SMEs) at the beginning of their life cycle, whereas a ladder of authority emerges as organizations grow and is likely common in established (large) organizations. In our view and in line with the new competitive setting that we described above, ladders of authority and of responsibility must coexist simultaneously rather than sequentially. As organizations grow, they should develop a top-down chain of command (ladder of authority) without stifling existing bottom-up ladders of responsibility. Although such coexistence can be challenging, it is a responsibility of the ladder of authority to develop simple guidelines and practices for the management of their interplay (Romme 2019).

The third question in Whetten’s model refers to ‘who’. All the OLC models converge in identifying the managers (and the owners) as the main actors in determining company development. We contend that in the contemporary business environment, particularly from the development of the stakeholder theory (Freeman et al. 2010), this view offers a limited perspective of the multitude of interacting actors who can affect the strategic objectives of the firms and, as a consequence, their growth and survival. For example, discussing stakeholder identification, Crane and Ruebottom (2011) propose a matrix counting over 60 possible actors. To understand how the companies’ growth and survival may be affected by new categories of stakeholders, we present some examples. In February 2018, with the rise of the #metoo movement, Guess cofounder Paul Marciano was accused of sexual harassment by a former model, and hours after the allegation (communicated via Twitter), the company’s shares dropped almost 18%. Again, according to a survey reported by the World Economic Forum (2020), companies increased their awareness of sustainability and energy issues because of the worldwide strikes inspired by Swedish activist Greta Thunberg. The multiplication of relevant stakeholders is reflected, among others, in the CEO’s average span of control, which, over the past two decades, has doubled, rising from about five in the mid-1980s to almost 10 in the mid-2000s (Neilson and Wulf 2012). As suggested by the authors, the increase in the chief executive’s direct reports is not surprising because companies today are vastly more complex, globally dispersed and strictly scrutinized than those of previous generations. Therefore, to manage such a vast array of actors potentially influencing their development, companies need to properly manage their horizontal differentiation. Following the well-known Ashby law of requisite variety (Ashby 1961), to thrive in a complex environment where a number of potentially contrasting but interdependent objectives arise, an organization must respond by increasing its level of internal complexity accordingly, that is, by creating new organizational units dedicated to a portion of the task environment and simultaneously increasing the level of integration among them.

Finally, addressing Whetten’s ‘how’ question, we showed that companies described in the traditional OLC models progress in their evolutionary process, solving crises and changing their internal organization. In particular, as in every stage of the model, the survival of the company is threatened by internal and external tensions, companies adopt different coordination mechanisms to maintain company manageability and prevent disruptive effects. Those mechanisms are aimed at solving the contrasting needs of larger and older companies. Considering company growth mainly as organic, the OLC models have failed to consider external strategies of growth, including mergers and acquisitions, strategic alliances or network forms. In all these circumstances, a firm sustains its growth by leveraging the combination of its own firm-specific capabilities with complementary knowledge of third-party sources. These modes of growth have become common over the years because of increasing competition, global dispersion of knowledge and the need for rapid new product development processes. For example, a recent survey in Europe on the trends in open innovation, corporate entrepreneurship and start-ups suggests that 97% of the innovation leaders interviewed will adopt co-development practices, and 90% will invest in start-ups (Mind the Bridge-Nesta 2019). The adoption of such practices shows the needs for using relevant coordination mechanisms because the outcome of external opportunities of growth is contingent on both the initial recognition (i.e., access to the resources and capabilities held by external sources) and its subsequent exploitation within the firm once resources have crossed organizational boundaries (Burton and Obel 1998; Jansen et al. 2005; Foss et al. 2011, 2013).

## Discussion

The OLC models provide a holistic approach toward firms’ organizational development over its life cycle. The fascinating power of their seminal idea which equates the growth of the company with the development of a person or plant, their comprehensive view on both the internal characteristics and the external conditions of a company, their focus on the simultaneous interactions of multiple elements, and their longitudinal perspective which is much needed since the speed of change and development is ever increasing and the research phenomena are far from static, represent the main reasons for their permanence in management studies (e.g., Kallunki and Silvola 2008; Wang and Singh 2014).

However, our analysis of their empirical validation and of their explanatory power in the actual business environment suggests several arguments against the continued use of OLC models in understanding organizations. Blurring industry boundaries, diminishing geographical barriers and pervasive new technologies make the distinction among stages seamless, while organizations can even leap-frog stages that they would have traditionally gone through. In addition, growth processes are endogenous and cannot be fully separated from the stages that the OLC models are supposed to explain. Finally, traditional OLC models underestimate the possible resistances associated with the change management process between one step and the other (Kotter 2012). Differently, firms face a variety of contrasting needs that make demands on their organizational structure. For instance, a company deals simultaneously with the need to maintain control and adaptation, efficiency and effectiveness, predictability of behaviors and innovation, agility and learning all while facing market volatility and continuing its development. Hence, organizational solutions must combine the choices usually considered in a trade-off, including, for example, the presence of self-managed teams and a line of authority, the adoption of formal practices and mutually evolving procedures or multiple horizontal differentiation logics.

Notwithstanding these limitations, we maintain the validity of the OLC perspective in today’s world but anchored on different assumptions. First, whereas *original* OLC models adopted growth in size as the engine of organizational evolution, *new* OLC models should move the focus to the environment to anticipate the need for organizational adaptation. Ageing companies should not be worried about their size to establish their level of organizational maturity, but instead, their development should be measured by their adaptations to the changing environmental conditions independently of growth. In particular, we advance the idea that the OLC models could be depicted as a sequence of organizational changes that represent life cycle turning points originating from different organizational alternatives. These alternatives represent temporary states of stability (i.e., what the original OLC models would call a ‘stage’) in the life cycle of a company: persistent change would increase the risks of role ambiguity and conflicts (Rivkin and Siggelkow 2003), hence threatening the survival of the company (Hannan and Freeman 1984). As a consequence, analyzing companies’ development according to an OLC perspective, we expect to recognize a sequence of temporary states of organizational stability (i.e., stages), but their duration and sequence cannot be generalized across different companies. Second, whereas the *original* OLC models proposed model organizational evolution according to a predetermined sequence of structural configurations, *new* OLC models should acknowledge that companies are characterized by a continuous search for a dynamic fit between environmental conditions and strategic and organizational choices (Soda and Furnari 2012), hence reducing the predictability of the resulting organizational features (Terziovski 2010; Brahm and Tarziján 2016). The outcome is not universal, but several organizational solutions are possible through an original combination of basic design elements (Sinha and Van de Ven 2005; Van de Ven et al. 2013). These solutions are *equifinal*, that is, they reach similar performance by means of different design options for a given environmental situation (Drazin and Van de Ven 1985; Gresov and Drazin 1997). Equifinality holds the idea that ‘a system can reach the same final state from different initial conditions and by a variety of different paths’ (Katz and Kahn 1978, p. 30). Our revision of OLC models suggests that organizational configurations are equifinal instead of universal either because organizational dimensions depend on one another or because organizational configurations must solve contrasting needs. Therefore, analyzing companies’ development according to an OLC perspective, we expect to *not* find similar sequences of organizational configurations and—*ceteris paribus* in terms of environmental situations—to find different (and equally effective) organizational structures.

## Conclusions, limitations and further research

In the current study, we aimed to build on the five primary elements of good theory (i.e., why, when, who, what and how) suggested by Whetten (1989) to analyze the organizational design characteristics of five seminal OLC models. We sustain that the OLC models propose a deterministic trajectory of organizational development that shows limited explanatory power when confronted with the challenges of today’s business environment. Conversely, we suggest conceiving OLC as a process where the ‘engine’ are the changes associated with the variety and uncertainty of the environment. Our argument is that OLC models preserve their *raison d'être* but on a different basis: organizations evolve somehow independently from their size following unpredictable paths that show a variety of equifinal configurations.

The present study has limitations that pave the way for future research opportunities. First, our analysis focuses on five theory-based OLC models because we purposely decided to consider only original OLC models based on intellectual sources that discuss how organizational design characteristics change in a firm’s life cycle. Future research may extend our review to include other OLC models dedicated to specific categories of firms, for example, entrepreneurial firms, new ventures or nonprofit organizations.

Second, we suggest what the organizational design consequences of a firm’s evolutionary process in today’s business environment could be. We hope that our arguments will stimulate future empirical validation. Specifically, empirical studies might want to test whether organizations adopt equifinal configurations at similar ages or under similar contingencies in similar industry settings, with the same strategies or under similar formal and informal institutions. Furthermore, organizational scholars may longitudinally explore equifinal organizational configurations and test to what extent such configurations are exogenously determined by the features of the environment in which they operate or endogenously conditioned by a path-dependent trajectory in which the evolutionary process is conditioned by past organizational configurations. A longitudinal study could also explore the transition from one change to another and the associated resistances. These research questions could be addressed by exploring novel research methodologies such as a qualitative comparative analysis (QCA) (Ragin 2009; Soda and Furnari 2012), experimentation and simulation (Davis et al. 2007; Burton and Obel 2011, 2018).
